# Network Pharmacology and Molecular Docking-Based Prediction of the Mechanism of Qianghuo Shengshi Decoction against Rheumatoid Arthritis

**DOI:** 10.1155/2021/6623912

**Published:** 2021-09-04

**Authors:** Zhihao Zeng, Jiaoting Hu, Jieyi Jiang, Guanlin Xiao, Ruipei Yang, Sumei Li, Yangxue Li, Huajing Huang, Huixian Zhong, Xiaoli Bi

**Affiliations:** ^1^School of the Fifth Clinical Medicine, Guangzhou University of Chinese Medicine, Guangzhou 510405, China; ^2^Artemisinin Research Center, Guangzhou University of Chinese Medicine, Guangzhou 510405, China; ^3^Guangdong Province Engineering and Technology Research Institute of Traditional Chinese Medicine, Guangzhou 510095, China; ^4^Guangdong Provincial Key Laboratory of Research and Development in Traditional Chinese Medicine, Guangzhou 510095, China

## Abstract

Qianghuo Shengshi decoction (QHSSD) is a classical Chinese medicine formula, which is used in clinical practice for the treatment of rheumatoid arthritis (RA) in China. However, the pharmacological mechanism of QHSSD on RA has remained unclear by now. We collected and screened active compounds and its potential targets by the pharmacology platform of Chinese herbal medicines. In addition, the therapeutic targets of RA were obtained and selected from databases. Network construction analyzed that 128 active compounds may act on 87 candidate targets and identified a total of 18 hub targets. GO annotation and KEGG enrichment investigated that the action mechanism underlying the treatment of RA by QHSSD might be involved in cell proliferation, angiogenesis, anti-inflammation, and antioxidation. Finally, molecular docking verification showed that TP53, VEGFA, TNF, EGFR, and NOS3 may be related to the RA treatment and molecular dynamics simulation showed the stability of protein-ligand interactions. In this work, QHSSD might exert therapeutic effect through a multicomponent, multitarget, and multipathway in RA from a holistic aspect, which provides basis for its mechanism of action and subsequent experiments.

## 1. Introduction

Rheumatoid arthritis (RA) is a chronic autoimmune disease with the following symptoms: tender, warm, swollen, and stiff joints in the morning and as RA progresses, resulting in bone erosion, joint deformity, and physical disabilities [1–4]. RA affects approximately 1% of the worldwide population and mostly occurs in middle-aged women [5]. The drug treatment of RA mainly focuses on nonsteroidal anti-inflammatory drugs (NSAIDs), steroids, disease-modifying antirheumatic drugs (DMARDs), and glucocorticoids. However, these drugs can only delay the progression of RA, which may also increase the risk of infection. Long-term use of these drugs will cause drug dependence and a cascade of side effects, such as digestive tract irritation reaction, rashes, liver toxicity, and hair loss [2, 6, 7].

There is a rising trend of the application of traditional Chinese medicine in the treatment of RA, and the RA patients are more inclined to choose complementary medicine as a medical treatment [8–10]. Traditional Chinese medicine (TCM) has a long history of clinical use in China, used as complementary and alternative medicines in treatment [11]. Qianghuo Shengshi decoction (QHSSD) is a classic clinical prescription from internal and external causes (*Nei Wai Shang Bian Huo Lun*). Accordingly, QHSSD can dispel wind and dampness and sweat and alleviate pain, consisting of seven herbs: Notopterygii rhizoma et radix (Qianghuo, QH), Angelicae pubescentis radix (Duhuo, DH), Saposhnikoviae radix (Fangfeng FF), Ligustici rhizoma et radix (Gaoben, GB), Chuanxiong rhizoma (Chuangxiong, CX), Viticis fructus (Manjingzi, MJZ), and Glycyrrhizae radix et rhizoma (Gancao, GC). QHSSD has been used to treat influenza, allergic purpura, and inflammatory diseases, such as RA [12–15]. Unlike the single-target agent with a clear mechanism, however, the mechanism of QHSSD underlying the treatment of RA still remains a blur.

**Figure 1:**
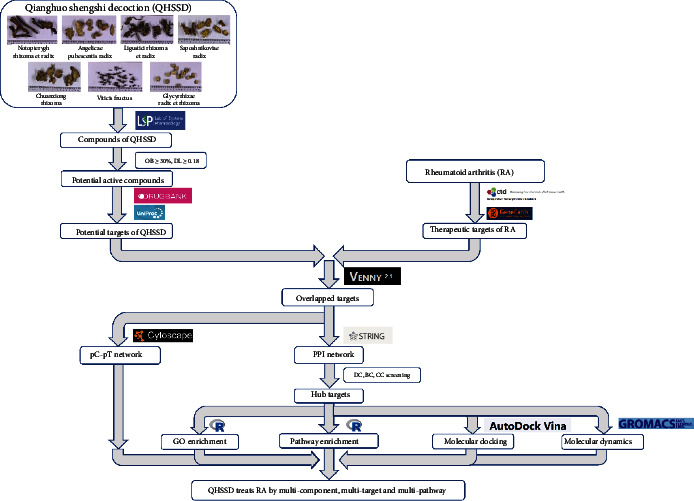
Flowchart of network pharmacology of QHSSD in the treatment of RA.

Network pharmacology is a novel approach to understand drug's pharmacological mechanism in the network perspective and to find out the interaction between the drug and the body on the basis of biological networks [16]. There is a growing number of researches that applied the network pharmacology to elucidate the mechanism between TCM/herbs and disease treatment, involving multicompound and multitarget ones [17–24]. We therefore tried to explore the mechanism of action of QHSSD on RA by network pharmacology and to further predict the interaction modes between QHSSD and the predicted targets by molecular docking and molecular dynamics. The flowchart of our study is presented in Figure 1.

## 2. Materials and Methods

### 2.1. Collecting and Screening of Active Components of QHSSD

We collected the chemical compounds of QHSSD from the traditional Chinese medicine systems pharmacology database and analysis platform (TCMSP) (https://tcmspw.com/tcmsp.php), a common tool for network pharmacology research of TCM [25]. Oral bioavailability (OB) and druglikeness (DL) were set as the main parameters to screen the active components according to the absorption, distribution, metabolism, and excretion (ADME) process. We set OB ≥ 30% and DL ≥ 0.18 as the screening threshold values to select the candidate components through literature search [23, 26, 27].

### 2.2. Potential Targets of QHSSD

Considering that the target screening criteria differ between different databases (including TCMSP, PubChem, and Swiss Target Prediction databases), therefore, only the TCMSP database was applied in this work. We predicted relevant targets (DrugBank, https://go.drugbank.com/) of active components in QHSSD on the TCMSP and then transformed the target name to a standard gene name on Uniport (https://www.uniprot.org/) database and removed the duplications.

### 2.3. Identification of Associated Targets of RA

RA-related genes were collected from the CTD (http://ctdbase.org/) database [28] and GeneCards (https://www.genecards.org/) database [29], with the keyword “rheumatoid arthritis.” The genes from the above databases merged and removed the duplications.

### 2.4. Compound-Target Network Construction and Analysis

Venny 2.1.0 (https://bioinfogp.cnb.csic.es/tools/venny/) [30] was used to find out the overlapping targets between compound targets and disease targets. To explore the relationship between compounds and disease more reasonably, Cytoscape 3.7.2 (https://cytoscape.org/) [31] with a visualized tool and all node degrees of networks calculated was used to construct a compound-potential target network.

### 2.5. Protein-Protein Interaction (PPI) Network Construction and Analysis

The Search Tool for the Retrieval of Interacting Genes/Proteins (STRING) (https://string-db.org/) was used to predict the interaction between proteins [32]. According to the previous steps, drug-associated genes and disease targets were analyzed on STRING to create a protein-protein interaction network (PPI network) by importing overlapping targets. Cytoscape was then used to construct a core PPI network.

### 2.6. Gene Ontology (GO) Enrichment and Kyoto Encyclopedia of Genes and Genomes (KEGG) Pathway Analysis

We imported the hub targets on the Bioconductor clusterProfiler org.Hs.eg.db and DOSE packages [33, 34] of R 4.0.2 (https://cran.r-project.org/src/base/R-4/) software to create the Kyoto Encyclopedia of Genes and Genomes (KEGG) pathway enrichment and construct Gene Ontology (GO) biological function enrichment analysis with *p* < 0.05 and *q* < 0.05 as the thresholds.

### 2.7. Compound-Target Molecular Docking

According to previous studies [19, 20], compounds with a higher degree value and potential targets were evaluated by molecular docking. The 3D structure format file of the active compounds screened from the previous step and the hub targets were downloaded from the PubChem (https://pubchem.ncbi.nlm.nih.gov/) database and the RCSB PDB (https://www.rcsb.org/), respectively. The active compounds are ligands and the targets are receptors. AutoDockTools 1.5.6 software was used to remove the water molecules, isolate proteins, add nonpolar hydrogen, and calculate Gasteiger charges for the structure. Both ligands and receptors were kept as PDBQT format docked through AutoDock Vina [35]. The Grid Box size and position of macromolecule were predicted by POCASA 1.1 (http://g6altair.sci.hokudai.ac.jp/g6/service/pocasa/). Then, the protein-ligand interaction was visualized with PyMOL 2.3.0 software.

### 2.8. Molecular Dynamics Simulations

Molecular dynamics (MD) simulations were used to evaluate the structural characteristics of the protein-ligand system [36]. In this work, the GROMACS Program package [37] and CHARMM36 force field [38] were used to simulate the molecular dynamics of the complexes in the TIP3P water model. Ions (Na^+^ or Cl^−^) were added to neutralize charges. The energy of systems was minimized through a 4000-step steepest descent method and a 2000-step conjugate gradient method to eliminate high-energy collisions between molecules. Then, the systems were heated from 0 K to 300 K within 100 ps and simulated as an NPT ensemble with normal temperature (300 K) and normal pressure (101 kPa) for 100 ps; unconstrained MD simulation was carried out for 10 ns after the system reached dynamic equilibrium. Periodic boundary conditions and particle mesh Ewald (PME) methods were used to deal with long-range electrostatic interactions. The short-range Coulomb interaction was 0.9 nm, and the SHAKE algorithm was used to restrict the bond to the hydrogen atom. The temperature and pressure of the system were controlled by V-rescale and Parrinello-Rahman. The stability of systems was evaluated using the root-mean-square deviation (RMSD) of the aligned protein-ligand coordinate set calculated against the initial frame.

## 3. Results

### 3.1. Compound-Target Network and Analysis

**Figure 2:**
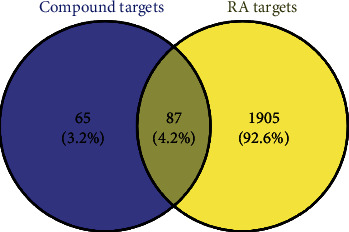
Venn diagram of overlapping targets of QHSSD and RA.

**Figure 3:**
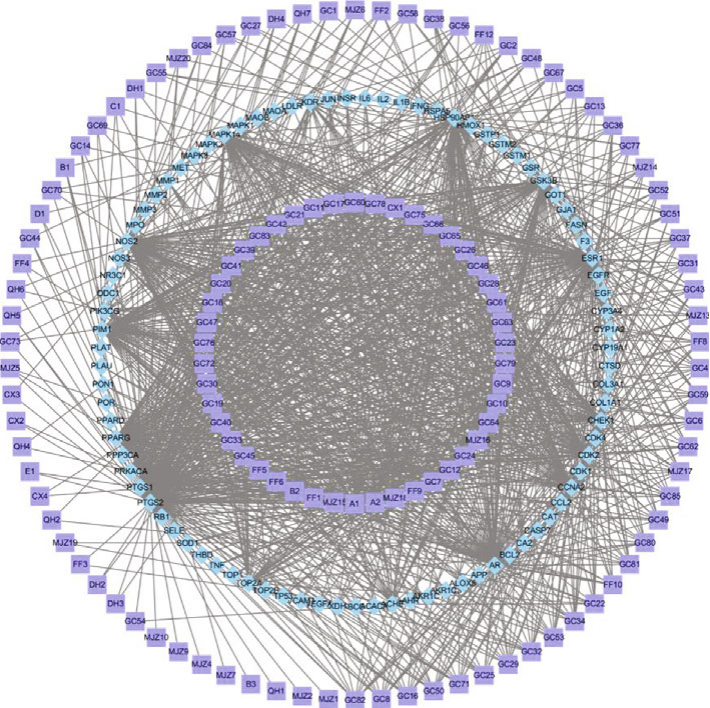
Compound-target network of QHSSD. The rectangle nodes–the compounds of herbs, the diamond nodes–the targets of compounds.

We collected 153 compounds and 152 putative targets of QHSSD (Table S1 and Figure S1) on the screening criteria from the TCMSP. Among these compounds, we excluded 25 compounds without RA-related targets and 128 active compounds were obtained in this work. 1846 targets were selected from 29368 genes related to RA by the screening threshold of inference score ≥ 24.46 on the CTD database. Meanwhile, 306 genes were obtained from 4465 targets with a relevance score ≥ 19.94 on GeneCards. Targets from the above database merged and removed the overlapping parts, and at last, we received a total of 1992 RA-related targets. Based on the Venn diagram, we obtained 87 overlapping targets from 152 putative targets of QHSSD and 1992 RA-related targets. Consequently, 87 targets (Figure 2 and Table 1) were considered as potential targets for the treatment. To further investigate the interaction between the compounds and targets, we constructed a compound-target network (Figure 3) with 215 nodes and 1239 edges. The network results manifested that the average degree value of the compounds was 9.68; we received 66 active compounds. Five compounds were considered to have high connection with putative targets of RA: quercetin (degree = 57), kaempferol (degree = 31), luteolin (degree = 27), wogonin (degree = 21), and isorhamnetin (degree = 20) (Table 2).

### 3.2. PPI Network and Analysis

**Figure 4:**
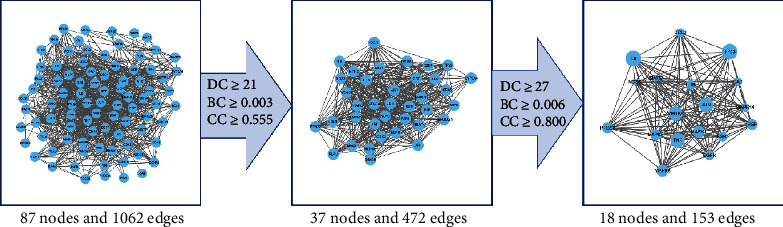
The screening process of the PPI network by Cytoscape (the larger nodes mean higher degree values).

Based on the previous results, 87 potential targets were imported in STRING with the organism set as “homo sapiens.” Those data with a confidence level higher than 0.4 constructed a complete PPI network including 87 nodes and 1062 edges. We chose three important parameters, including “degree (DC),” “betweenness centrality (BC),” and “closeness centrality (CC),” as the thresholds to screen the key genes to establish major hub nodes by Cytoscape. First, we set the medium of DC, BC, and CC as thresholds, DC ≥ 21, BC ≥ 0.003, and CC ≥ 0.555, respectively, and obtained 37 nodes and 472 edges. Then, the 37 key nodes were further filtered by the second threshold of DC ≥ 27, BC ≥ 0.006, and CC ≥ 0.800 and we got 18 nodes and 153 edges at last (Figure 4). The hub targets of QHSSD in RA therapy are as follows: IL-6, TP53, VEGFA, JUN, TNF, MAPK1, MAPK8, EGF, PTGS2, MAPK3, EGFR, ESR1, IL-1B, CAT, CCL2, MAPK14, MMP2, and NOS3.

### 3.3. GO and KEGG Pathway Enrichment Analysis

**Figure 5:**
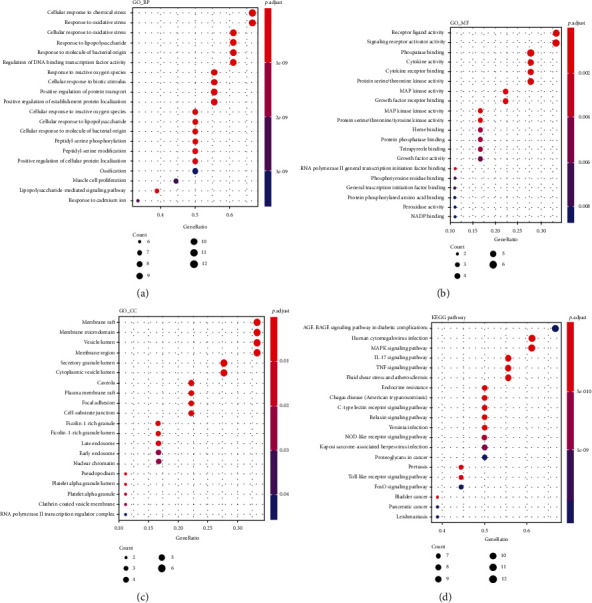
Enrichment analysis of potential targets. (a) GO biological process analysis, (b) GO molecular function, (c) GO cellular component, and (d) KEGG pathway analysis.

We performed GO and KEGG pathways to further elucidate the biological functions of 18 hub targets systematically. There were 1534 GO items (1474 terms of biological process, 40 terms of molecular function, and 20 terms of cellular component) and 142 pathways of KEGG enrichment. The top 20 GO items and KEGG pathways are displayed in Figure 5. The biological processes of targets were mainly enriched in oxidative response/stress (GO: 0034599, GO: 0006979, GO: 0000302, and GO: 0034614), inflammatory response (GO: 0032496, GO: 0031663, and GO: 0071222), and biochemical stimulation (GO: 0062197, GO: 0002237, GO: 0071216, and GO: 0071219). Additionally, the KEGG pathways were most enriched in immune- and inflammatory-associated pathways, including the MAPK signaling pathway (hsa04010), IL-17 signaling pathway (hsa04657), TNF signaling pathway (hsa04668), Toll-like receptor signaling pathway (hsa04620), and C-type lectin receptor signaling pathway (hsa04625), and involved in angiogenesis and atherosclerosis pathways: fluid shear stress and atherosclerosis (hsa05418) and relaxin signaling pathway (hsa04926). The compounds of QHSSD may act on these signaling pathways and yield a therapeutic effect.

### 3.4. Molecular Docking Analysis

To further elucidate the mechanism of action, 18 hub targets with their related compounds were docked and screened by the binding affinity on AutoDock Vina based on the compound-target network. We investigated a total of 39 pairs of ligand-protein interaction (Table 3), with binding affinity < −5 kcal/mol as the screening threshold. The lower the value of the docking affinity, the stronger the binding ability between the compound and the active site of the target. Accordingly, JUN and TNF get a low binding ability with their related compounds, as well as JUN-quercetin, JUN-kaempferol, and TNF-quercetin. Therefore, 3 pairs of combinations were excluded, and at last, we retrieved 47 compound-target pairs. In addition, cognate docking was used to verify the rationality of molecular docking. In order to verify the rationality of the docking for the TP53 and quercetin system, we investigated the docking of IRAK4 (PDB ID: 2a9i) and quercetin; the binding affinity of IRAK4-quercetin was −6.6 kcal/mol. Small-molecule ligand potentially could fit into the interface port formed by the interaction of amino acid residues in protein. Overall, these complexes provide further evidence that these proteins could act as therapeutic target for the compound of QHSSD.

### 3.5. Molecular Dynamics Simulations

**Figure 6:**
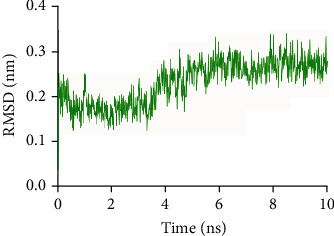
RMSD change of TP53 backbone atoms in MD simulation.

**Figure 7:**
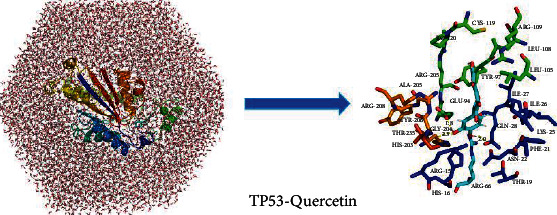
Detailed view of hydrogen bonds between TP53 and quercetin.

To further research the stability of the molecular dynamics trajectory, we took a complex (TP53-quercetin) as an example to analyze the root mean square deviation (RMSD) of the TP53 backbone atoms in this complex, as shown in Figure 6. The RMSD of the TP53 backbone atom began to show an upward trend, whose trajectory was stable and reached an equilibrium state. The RMSD remained stable at about 0.2 nm after 4 ns, but in 7 ns, the trajectory fluctuated slightly. In addition, after 2 ns of the molecular dynamics simulation, the potential energy of the system also tends to be the minimum and stabilizes. The total interaction energy value was −136.2 ± 19.0 kJ/mol. The 4 ns simulated trajectory was extracted by GROMACS as the representative frame of the interaction between quercetin and TP53 (Figure 7); we could find that the amino acid ARG15 of TP53 and quercetin have hydrophobic interactions, resulting in quercetin and TP53 more closely. Moreover, quercetin and TP53 could form three hydrogen bonds (the hydrogen bond between the oxygen atom of quercetin and the hydrogen atom of amino acid residue GLY204 and the hydrogen bond between the hydrogen atom of quercetin and the nitrogen atom of amino acid residues GLU209 and GLN28) and caused the complex to bind more tightly.

## 4. Discussion

There has been increased researches on how traditional Chinese herbal medicine or formulae work on various diseases based on network pharmacology [21, 22, 24]. In our study, we first collected the putative targets of compounds in QHSSD from the database. And then, the compound-target network was constructed, which revealed that one herb could correspond with multiple components and multiple targets. The traditional Chinese medicine integrative database (TCMID) and a Bioinformatics Analysis Tool for Molecular Mechanism of Traditional Chinese Medicine (BATMAN-TCM) database were used to predict the targets of Chinese herbal medicine [27, 28], whereas we found that the results of these databases were different because of the screening criteria. To ensure the consistency of data, we only used the TCMSP database to collect active compounds and related targets. In compound-target network analysis, the top five compounds (quercetin, kaempferol, luteolin, wogonin, and isorhamnetin) were considered to be crucial compounds in QHSSD. Surprisingly, these compounds are both flavonoids which have antioxidant and anti-inflammatory effects [39–42]. For instance, researches indicated that quercetin mediate the anti-inflammatory mechanism of reducing acute laryngitis by restoring the Th17/Treg balance and activating heme oxygenase 1 and isorhamnetin increased the levels of TNF-*α*, IL-1*β*, IL-6, IL-17A, and IL-17F as well as decreased levels of IL-35 and IL-10 in the CIA mice, resulting in an anti-inflammatory effect [43, 44]. Luteolin prevented cartilage destruction in OA rats in vivo through inhibiting IL-1*β*-induced phosphorylation of NF-*κ*B [45]. In conclusion, these active compounds are crucial material basis of the potential therapeutic effect of QHSSD on RA.

Further, we selected 18 hub targets from the PPT network, as follows: IL-6, TP53, VEGFA, JUN, TNF, MAPK1, MAPK8, EGF, PTGS2, MAPK3, EGFR, ESR1, IL-1B, CAT, CCL2, MAPK14, MMP2, and NOS3. Combined with GO annotation and pathway enrichment analysis, we preliminarily considered that the treatment of RA by QHSSD may participate in biological processes of proliferation, inflammatory response, and angiogenesis. Previous studies indicated that IL-6 and TNF antagonists decrease the inflammatory response of RA patients and IL-1B stimulates inflammation and degradation of the bone and cartilage [46–49]. EGF is an important growth factor, regulating the proliferation of cells. MAPK enables the growth and proliferation of cells by binding with other protein kinases or factors [50]. In addition, the formation of new blood vessels supplied nutrients and oxygen to the augmented inflammatory cell mass, contributing to perpetuation of joint disease [51]. VEGFA, a proangiogenic molecule, promotes the angiogenic phenotype of RA and is upregulated in RA [52, 53]. These literatures are consistent with our prediction. Therefore, these hub targets are considered as crucial proteins in RA therapy.

To further verify the hypothesis of action mechanism, we performed molecular docking on the hub target binding with crucial compounds. Surprisingly, we found that quercetin, luteolin, and wogonin could both fitly bind with TP53, a transcription factor, regulating cell cycle. Moreover, quercetin and luteolin also act on VEGFA. Previous studies revealed that TP53 mutations are linked to the VEGF pathway and showed that the transcriptional target of TP53 inhibited proliferation and angiogenesis via VEGFA [54–56]. We therefore hypothesized that quercetin and luteolin positively worked on TP53 to regulate cell cycle, and meanwhile act on VEGFA to suppress vascular formation. Additionally, luteolin could simultaneously act on EGFR and TNF, decreasing cell proliferation and the level of proinflammatory cytokines. This is in line with the researches before; the result of which manifested that luteolin inhibited IL-1*β*-induced expression of NO, PGE2, TNF-*α*, and MMP-2 [45, 50, 57, 58]. On the one hand, the synergistic action of quercetin and luteolin produces the effect of inhibiting cell proliferation and angiogenesis by multiple proteins and also has an anti-inflammatory effect. On the other hand, kaempferol acting on NOS3 may increase NO levels and prevent oxidative damage [59]. From a holistic aspect, QHSSD could inhibit the cell proliferation and angiogenesis and yield anti-inflammatory and antioxidant effects through regulating TP53, VEGFA, EGFR, TNF, and NOS3.

MD is crucial to the discovery of new drug [60] and is a powerful method for studying protein-ligand interactions [61]. In contrast to experimental methods alone, MD simulations can address diseases caused by protein misfolding and virtual screening; it also identifies the stability of protein-ligand complexes and ligand binding kinetics [62]. Previous studies found that quercetin can play an important role in inflammation by acting on the TP53 target [54–56]. In our study, MD simulation was used to investigate the interactions of TP53-quercetin. This complex was simulated within 10 ns and reached an equilibrium state. We found that ligand has three hydrogen bonds with protein; a study [63] had shown that hydrogen bonding has an advantage over shapeless forces if it can form hydrogen bonds. In this study, MD simulation revealed that the structure of TP53-quercetin was very stable when the simulation temperature was 300 K. It is important to explore the application of new drug by molecular docking and MD simulations [61]; however, it is still necessary to perform in vitro experiment to verify the prediction.

## 5. Conclusion

To conclude, we could learn that multicompounds act on multitargets in TCM therapy. We speculated that the therapeutic effect on the treatment of RA by QHSSD may be focused on antiangiogenesis, meanwhile, anti-inflammation, especially TP53 and VEGFA targets, plays a vital role during the treatment. This study was based on the combination of network pharmacology and molecular docking to predict and identify the mechanism of QHSSD in the treatment of RA, which provides the foundation for subsequent experimental investigation and offers ideas for the multidimensional and multilevel research of TCM formulae.

## Figures and Tables

**Table 1:** Information of 87 potential targets.

No.	Target name	Gene name	Gene ID
T1	Prostaglandin G/H synthase 2	PTGS2	5743
T2	Estrogen receptor	ESR1	2099
T3	Heat shock protein HSP 90-beta	HSP90AB1	3326
T4	Androgen receptor	AR	367
T5	Nitric oxide synthase, inducible	NOS2	4843
T6	Serine/threonine-protein kinase pim-1	PIM1	5292
T7	Cyclin-dependent kinase 2	CDK2	1017
T8	Peroxisome proliferator-activated receptor gamma	PPARG	5468
T9	Glycogen synthase kinase-3 beta	GSK3B	2932
T10	Prostaglandin G/H synthase 1	PTGS1	5742
T11	Cyclin-A2	CCNA2	890
T12	Mitogen-activated protein kinase 14	MAPK14	1432
T13	Serine/threonine-protein kinase Chk1	CHEK1	1111
T14	cAMP-dependent protein kinase catalytic subunit alpha	PRKACA	5566
T15	Acetylcholinesterase	ACHE	43
T16	DNA topoisomerase 2-alpha	TOP2A	7153
T17	Phosphatidylinositol 4,5-bisphosphate 3-kinase catalytic subunit gamma isoform	PIK3CG	5294
T18	Vascular endothelial growth factor receptor 2	KDR	3791
T19	Nitric oxide synthase, endothelial	NOS3	4846
T20	Amine oxidase	MAOB	4129
T21	Carbonic anhydrase 2	CA2	760
T22	Apoptosis regulator Bcl-2	BCL2	596
T23	Transcription factor AP-1	JUN	3725
T24	DNA topoisomerase 2-beta	TOP2B	7155
T25	Interstitial collagenase	MMP1	4312
T26	Tumor necrosis factor	TNF	7124
T27	Xanthine dehydrogenase/oxidase	XDH	7498
T28	Glutathione S-transferase P	GSTP1	2950
T29	Mitogen-activated protein kinase 1	MAPK1	5594
T30	Cellular tumor antigen p53	TP53	7157
T31	Interleukin-6	IL6	3569
T32	Glucocorticoid receptor	NR3C1	2908
T33	Heme oxygenase 1	HMOX1	3162
T34	Insulin receptor	INSR	3643
T35	Retinoblastoma-associated protein	RB1	5925
T36	Superoxide dismutase	SOD1	6647
T37	Serum paraoxonase/arylesterase 1	PON1	5444
T38	C-C motif chemokine 2	CCL2	6347
T39	Cyclin-dependent kinase 1	CDK1	983
T40	Cytochrome P450 3A4	CYP3A4	1576
T41	Cytochrome P450 1A2	CYP1A2	1544
T42	E-selectin	SELE	6401
T43	Vascular cell adhesion protein 1	VCAM1	7412
T44	Arachidonate 5-lipoxygenase	ALOX5	240
T45	Aryl hydrocarbon receptor	AHR	196
T46	Glutathione S-transferase Mu 1	GSTM1	2944
T47	Glutathione S-transferase Mu 2	GSTM2	2946
T48	Urokinase-type plasminogen activator	PLAU	5328
T49	Epidermal growth factor receptor	EGFR	1956
T50	Vascular endothelial growth factor A	VEGFA	7422
T51	72 kDa type IV collagenase	MMP2	4313
T52	Cyclin-dependent kinase 4	CDK4	1019
T53	DNA topoisomerase 1	TOP1	7150
T54	Interleukin-2	IL2	3558
T55	Interferon gamma	IFNG	3458
T56	Peroxisome proliferator-activated receptor delta	PPARD	5467
T57	Mitogen-activated protein kinase 8	MAPK8	5599
T58	Serine/threonine-protein phosphatase 2B catalytic subunit alpha isoform	PPP3CA	5530
T59	Aldo-keto reductase family 1 member C3	AKR1C3	8644
T60	Amine oxidase	MAOA	4128
T61	Amyloid-beta precursor protein	APP	351
T62	Caspase-7	CASP7	840
T63	Hepatocyte growth factor receptor	MET	4233
T64	Stromelysin-1	MMP3	4314
T65	Pro-epidermal growth factor	EGF	1950
T66	NADPH--cytochrome P450 reductase	POR	5447
T67	Ornithine decarboxylase	ODC1	4953
T68	Endoplasmic reticulum chaperone BiP	HSPA5	3309
T69	Acetyl-CoA carboxylase 1	ACACA	31
T70	Tissue factor	F3	2152
T71	Gap junction alpha-1 protein	GJA1	2697
T72	Interleukin-1 beta	IL1B	3553
T73	Tissue-type plasminogen activator	PLAT	5327
T74	Thrombomodulin	THBD	7056
T75	Collagen alpha-1	COL1A1	1277
T76	Myeloperoxidase	MPO	4353
T77	Collagen alpha-1	COL3A1	1281
T78	Cathepsin D	CTSD	1509
T79	Mitogen-activated protein kinase 3	MAPK3	5595
T80	Fatty acid synthase	FASN	2194
T81	Low-density lipoprotein receptor	LDLR	3949
T82	Catalase	CAT	847
T83	Aromatase	CYP19A1	1588
T84	Glutathione reductase, mitochondrial	GSR	2936
T85	Multidrug resistance-associated protein 1	ABCC1	4363
T86	Aldo-keto reductase family 1 member C1	AKR1C1	1645
T87	Aspartate aminotransferase, cytoplasmic	GOT1	2805

**Table 2:** Information of the top five active compounds of QHSSD.

No.	Mol ID	Chemical component	Degree	CAS	Herb
A1	MOL000098	Quercetin	57	117-39-5	MJZ/GC
A2	MOL000422	Kaempferol	31	520-18-3	MJZ/GC
MJZ18	MOL000006	Luteolin	27	491-70-3	MJZ
FF9	MOL000173	Wogonin	21	632-85-9	FF
GC7	MOL000354	Isorhamnetin	20	480-19-3	GC

QHSSD: Qianghuo Shengshi decoction; FF: Fangfeng; MJZ: Manjingzi; GC: Gancao.

**Table 3:** Results of 18 hub targets and related compounds of molecular docking.

No.	Targets	PDB ID	Compound	Binding affinity (kcal/mol)
1	IL6	4O9H	Quercetin	−6.4
			Luteolin	−6.4
			Wogonin	−6.4
2	TP53	3DCY	Quercetin	−7.2
			Luteolin	−7.5
			Wogonin	−7.7
3	VEGFA	5DN2	Quercetin	−8.1
			Luteolin	−8.1
4	JUN	1JUN	Quercetin	−4.8
			Kaempferol	−4.9
			Luteolin	−5.0
			Wogonin	−5.0
5	TNF	1TNF	Quercetin	−4.9
			Kaempferol	−5.3
			Luteolin	−5.4
			Wogonin	−5.3
6	MAPK1	6RSM	Quercetin	−8.3
			Luteolin	−8.5
			Naringenin	−8.2
7	MAPK8	4YR8	Kaempferol	−8.6
8	EGF	2KV4	Quercetin	−6.7
9	PTGS2	5F19	Quercetin	−9.4
			Kaempferol	−8.9
			Luteolin	−9.8
			Wogonin	−9.0
			Isorhamnetin	−8.8
			Hesperetin	−10.2
			Glepidotin B	−9.3
			Naringenin	−9.3
10	MAPK3	2ZOQ	Naringenin	−8.7
11	EGFR	4R3P	Quercetin	−7.3
			Luteolin	−7.5
12	ESR1	4XI3	Wogonin	−7.2
			Isorhamnetin	−7.6
			Hesperetin	−7.5
			Glepidotin B	−6.8
			Naringenin	−8.0
13	IL1B	5BVP	Quercetin	−5.7
14	CAT	1DGH	Naringenin	−7.9
15	CCL2	4ZK9	Quercetin	−7.7
			Wogonin	−6.9
16	MAPK14	3MGY	Wogonin	−7.6
			Isorhamnetin	−8.0
			Hesperetin	−8.2
17	MMP2	1EAK	Quercetin	−7.2
			Luteolin	−7.6
18	NOS3	1M9Q	Quercetin	−9.4
			Kaempferol	−8.5
			Isorhamnetin	−8.5
			Glepidotin B	−8.4

## Data Availability

The data used to support the findings of this study are included within the supplementary information files.

## Supplementary Materials

Supplementary Materials Table S1: information of active compounds of QHSSD. Figure S1: herb-compound-target network of QHSSD. (Supplementary Materials).
