# Supplementary material for: Network Pharmacology and Molecular Docking-Based Prediction of the Mechanism of Qianghuo Shengshi Decoction against Rheumatoid Arthritis
Source: Biomed Res Int. 2021 Sep 4;2021:6623912. doi: 10.1155/2021/6623912 (PMC8437630; doi:10.1155/2021/6623912)
Supplement: Supplementary Materials — Table S1: information of active compounds of QHSSD. Figure S1: herb-compound-target network of QHSSD. (Supplementary Materials). [file 6623912.f1.docx]

TABLE S1: Information of active compounds of QHSSD

| No. | Mol ID | Chemical component | OB/% | DL | Herb |
| --- | --- | --- | --- | --- | --- |
| B1 | MOL001941 | Ammidin | 34.55 | 0.22 | QH/DH/FF |
| - | MOL011962 | 6'-Feruloylnodakenin | 32.02 | 0.67 | QH |
| QH1 | MOL011963 | 8-geranoxy-5-methoxypsoralen | 40.97 | 0.50 | QH |
| - | MOL011968 | coumarin,glycoside | 33.07 | 0.78 | QH |
| QH2 | MOL011969 | Demethylfuropinnarin | 41.31 | 0.21 | QH |
| QH3 | MOL011971 | Diversoside_qt | 67.57 | 0.31 | QH |
| QH4 | MOL011975 | Notoptol | 62.97 | 0.48 | QH |
| QH5 | MOL001951 | Bergaptin | 41.73 | 0.42 | QH |
| QH6 | MOL001956 | Cnidilin | 32.69 | 0.28 | QH |
| F1 | MOL000359 | Sitosterol | 36.91 | 0.75 | QH/FF/GB/CX/MJZ/GC |
| C1 | MOL004792 | Nodakenin | 57.12 | 0.69 | QH/DH |
| B2 | MOL000358 | Beta-sitosterol | 36.91 | 0.75 | QH/DH/FF |
| B3 | MOL001942 | Isoimperatorin | 45.46 | 0.23 | QH/DH/FF |
| D1 | MOL002644 | Phellopterin | 40.19 | 0.28 | QH/FF |
| QH7 | MOL002881 | Diosmetin | 31.14 | 0.27 | QH |
| DH1 | MOL003608 | O-Acetylcolumbianetin | 60.04 | 0.26 | DH |
| DH2 | MOL004777 | Angelol D | 34.85 | 0.34 | DH |
| DH3 | MOL004778 | [(1R,2R)-2,3-dihydroxy-1-(7-methoxy-2-oxochromen-6-yl)-3-methylbutyl] (Z)-2-methylbut-2-enoate | 46.03 | 0.34 | DH |
| DH4 | MOL004780 | Angelicone | 30.99 | 0.19 | DH |
| - | MOL004782 | [(1R,2R)-2,3-dihydroxy-1-(7-methoxy-2-oxochromen-6-yl)-3-methylbutyl] 3-methylbutanoate | 45.19 | 0.34 | DH |
| FF1 | MOL000011 | (2R,3R)-3-(4-hydroxy-3-methoxy-phenyl)-5-methoxy-2-methylol-2,3-dihydropyrano[5,6-h][1,4]benzodioxin-9-one | 68.83 | 0.66 | FF |
| FF2 | MOL011730 | 11-hydroxy-sec-o-beta-d-glucosylhamaudol_qt | 50.24 | 0.27 | FF |
| FF3 | MOL011732 | Anomalin | 59.65 | 0.66 | FF |
| FF4 | MOL011737 | Divaricatacid | 87.00 | 0.32 | FF |
| FF5 | MOL011740 | Divaricatol | 31.65 | 0.38 | FF |
| FF6 | MOL011747 | Ledebouriellol | 32.05 | 0.51 | FF |
| FF7 | MOL011749 | Phelloptorin | 43.39 | 0.28 | FF |
| FF8 | MOL011753 | 5-O-Methylvisamminol | 37.99 | 0.25 | FF |
| FF9 | MOL000173 | Wogonin | 30.68 | 0.23 | FF |
| E1 | MOL001494 | Mandenol | 42.00 | 0.19 | FF/CX |
| FF10 | MOL003588 | Prangenidin | 36.31 | 0.22 | FF |
| FF11 | MOL007514 | Methyl icosa-11,14-dienoate | 39.67 | 0.23 | FF |
| FF12 | MOL013077 | Decursin | 39.27 | 0.38 | FF |
| CX1 | MOL002135 | Myricanone | 40.60 | 0.51 | CX |
| CX2 | MOL002140 | Perlolyrine | 65.95 | 0.27 | CX |
| - | MOL002151 | Senkyunone | 47.66 | 0.24 | CX |
| CX3 | MOL002157 | Wallichilide | 42.31 | 0.71 | CX |
| CX4 | MOL000433 | FA | 68.96 | 0.71 | CX |
| MJZ1 | MOL011901 | (2E)-2-[(1R,2R,4aS,8aS)-2,5,5,8a-tetramethylspiro[decalin-1,5'-tetrahydrofuran]-2'-ylidene]acetaldehyde | 54.24 | 0.22 | MJZ |
| MJZ2 | MOL011903 | 12s,16s(r)-dihydroxy-ent-labda-7,13-dien-15,16-olide | 37.98 | 0.31 | MJZ |
| MJZ3 | MOL011906 | Acetic acid [(1R,3R,4R,4aS,8aS)-4-hydroxy-3,4a,8,8-tetramethyl-4-[2-(2-oxo-5H-furan-3-yl)ethyl]-1-decalinyl] ester | 59.38 | 0.39 | MJZ |
| - | MOL011909 | (3R)-5-[(1S,4aS,8aS)-5,5,8a-trimethyl-2-methylene-1-decalinyl]-3-methylpent-1-en-3-ol | 46.08 | 0.18 | MJZ |
| MJZ4 | MOL011910 | Rimuene | 35.73 | 0.22 | MJZ |
| MJZ5 | MOL011912 | (2R,4aS,10aR)-7-isopropyl-2,4a-dimethyl-1-methylene-4,9,10,10a-tetrahydro-3H-phenanthren-2-ol | 48.61 | 0.25 | MJZ |
| MJZ6 | MOL007274 | Skrofulein | 30.35 | 0.30 | MJZ |
| MJZ7 | MOL011923 | Previtexilactone | 45.09 | 0.45 | MJZ |
| MJZ8 | MOL011929 | Vitetrifolin c | 63.84 | 0.35 | MJZ |
| MJZ9 | MOL011930 | Vitetrifolin d | 40.42 | 0.39 | MJZ |
| MJZ10 | MOL011931 | Vitetrifolin e | 31.41 | 0.30 | MJZ |
| - | MOL011934 | Vitexifolin A | 32.75 | 0.18 | MJZ |
| MJZ11 | MOL011935 | Acetic acid [(1R,3R,4R,4aS,8aS)-4-hydroxy-3,4a,8,8-tetramethyl-4-[2-(5-oxo-2H-furan-3-yl)ethyl]-1-decalinyl] ester | 57.69 | 0.39 | MJZ |
| MJZ12 | MOL011937 | acetic acid [(1R,3R,4R,4aS,8aS)-4-hydroxy-4-[2-(2-keto-3-pyrrolin-3-yl)ethyl]-3,4a,8,8-tetramethyl-decalin-1-yl] ester | 50.49 | 0.39 | MJZ |
| MJZ13 | MOL011938 | 4-(3,4-dimethoxyphenyl)-6-hydroxy-5-methoxynaphthalene-2-carbaldehyde | 79.17 | 0.36 | MJZ |
| - | MOL011939 | Vitrofolal B | 41.46 | 0.40 | MJZ |
| MJZ14 | MOL011940 | Vitrofolal C | 42.20 | 0.66 | MJZ |
| A2 | MOL000422 | Kaempferol | 41.88 | 0.24 | MJZ/GC |
| MJZ15 | MOL000449 | Stigmasterol | 43.83 | 0.76 | MJZ |
| MJZ16 | MOL005100 | Hesperetin | 47.74 | 0.27 | MJZ |
| MJZ17 | MOL005229 | Artemetin | 49.55 | 0.48 | MJZ |
| MJZ18 | MOL000006 | Luteolin | 36.16 | 0.25 | MJZ |
| - | MOL011169 | Peroxyergosterol | 44.39 | 0.82 | MJZ |
| MJZ19 | MOL007107 | C09092 | 36.07 | 0.25 | MJZ |
| A1 | MOL000098 | Quercetin | 46.43 | 0.28 | MJZ/GC |
| MJZ20 | MOL004576 | Taxifolin | 57.84 | 0.27 | MJZ |
| GC1 | MOL001484 | Inermine | 75.18 | 0.54 | GC |
| GC2 | MOL001792 | DFV | 32.76 | 0.18 | GC |
| GC3 | MOL000211 | Mairin | 55.38 | 0.78 | GC |
| GC4 | MOL002311 | Glycyrol | 90.78 | 0.67 | GC |
| GC5 | MOL000239 | Jaranol | 50.83 | 0.29 | GC |
| GC6 | MOL002565 | Medicarpin | 49.22 | 0.34 | GC |
| GC7 | MOL000354 | Isorhamnetin | 49.60 | 0.31 | GC |
| GC8 | MOL003656 | Lupiwighteone | 51.64 | 0.37 | GC |
| GC9 | MOL003896 | 7-Methoxy-2-methyl isoflavone | 42.56 | 0.2 | GC |
| GC10 | MOL000392 | Formononetin | 69.67 | 0.21 | GC |
| GC11 | MOL000417 | Calycosin | 47.75 | 0.24 | GC |
| GC12 | MOL004328 | Naringenin | 59.29 | 0.21 | GC |
| GC13 | MOL004805 | (2S)-2-[4-hydroxy-3-(3-methylbut-2-enyl)phenyl]-8,8-dimethyl-2,3-dihydropyrano[2,3-f]chromen-4-one | 31.79 | 0.72 | GC |
| GC14 | MOL004806 | Euchrenone | 30.29 | 0.57 | GC |
| GC15 | MOL004808 | Glyasperin B | 65.22 | 0.44 | GC |
| GC16 | MOL004810 | Glyasperin F | 75.84 | 0.54 | GC |
| GC17 | MOL004811 | Glyasperin C | 45.56 | 0.4 | GC |
| GC18 | MOL004814 | Isotrifoliol | 31.94 | 0.42 | GC |
| GC19 | MOL004815 | (E)-1-(2,4-dihydroxyphenyl)-3-(2,2-dimethylchromen-6-yl)prop-2-en-1-one | 39.62 | 0.35 | GC |
| GC20 | MOL004820 | Kanzonols W | 50.48 | 0.52 | GC |
| GC21 | MOL004824 | (2S)-6-(2,4-dihydroxyphenyl)-2-(2-hydroxypropan-2-yl)-4-methoxy-2,3-dihydrofuro[3,2-g]chromen-7-one | 60.25 | 0.63 | GC |
| GC22 | MOL004827 | Semilicoisoflavone B | 48.78 | 0.55 | GC |
| GC23 | MOL004828 | Glepidotin A | 44.72 | 0.35 | GC |
| GC24 | MOL004829 | Glepidotin B | 64.46 | 0.34 | GC |
| GC25 | MOL004833 | Phaseolinisoflavan | 32.01 | 0.45 | GC |
| GC26 | MOL004835 | Glypallichalcone | 61.60 | 0.19 | GC |
| GC27 | MOL004838 | 8-(6-hydroxy-2-benzofuranyl)-2,2-dimethyl-5-chromenol | 58.44 | 0.38 | GC |
| GC28 | MOL004841 | Licochalcone B | 76.76 | 0.19 | GC |
| GC29 | MOL004848 | Licochalcone G | 49.25 | 0.32 | GC |
| GC30 | MOL004849 | 3-(2,4-dihydroxyphenyl)-8-(1,1-dimethylprop-2-enyl)-7-hydroxy-5-methoxy-coumarin | 59.62 | 0.43 | GC |
| GC31 | MOL004855 | Licoricone | 63.58 | 0.47 | GC |
| GC32 | MOL004856 | Gancaonin A | 51.08 | 0.4 | GC |
| GC33 | MOL004857 | Gancaonin B | 48.79 | 0.45 | GC |
| - | MOL004860 | Licorice glycoside E | 32.89 | 0.27 | GC |
| GC34 | MOL004863 | 3-(3,4-dihydroxyphenyl)-5,7-dihydroxy-8-(3-methylbut-2-enyl)chromone | 66.37 | 0.41 | GC |
| GC35 | MOL004864 | 5,7-dihydroxy-3-(4-methoxyphenyl)-8-(3-methylbut-2-enyl)chromone | 30.49 | 0.41 | GC |
| GC36 | MOL004866 | 2-(3,4-dihydroxyphenyl)-5,7-dihydroxy-6-(3-methylbut-2-enyl)chromone | 44.15 | 0.41 | GC |
| GC37 | MOL004879 | Glycyrin | 52.61 | 0.47 | GC |
| GC38 | MOL004882 | Licocoumarone | 33.21 | 0.36 | GC |
| GC39 | MOL004883 | Licoisoflavone | 41.61 | 0.42 | GC |
| GC40 | MOL004884 | Licoisoflavone B | 38.93 | 0.55 | GC |
| GC41 | MOL004885 | Licoisoflavanone | 52.47 | 0.54 | GC |
| GC42 | MOL004891 | Shinpterocarpin | 80.30 | 0.73 | GC |
| GC43 | MOL004898 | (E)-3-[3,4-dihydroxy-5-(3-methylbut-2-enyl)phenyl]-1-(2,4-dihydroxyphenyl)prop-2-en-1-one | 46.27 | 0.31 | GC |
| GC44 | MOL004903 | Liquiritin | 65.69 | 0.74 | GC |
| GC45 | MOL004904 | Licopyranocoumarin | 80.36 | 0.65 | GC |
| - | MOL004905 | 3,22-Dihydroxy-11-oxo-delta(12)-oleanene-27-alpha-methoxycarbonyl-29-oic acid | 34.32 | 0.55 | GC |
| GC46 | MOL004907 | Glyzaglabrin | 61.07 | 0.35 | GC |
| GC47 | MOL004908 | Glabridin | 53.25 | 0.47 | GC |
| GC48 | MOL004910 | Glabranin | 52.90 | 0.31 | GC |
| GC49 | MOL004911 | Glabrene | 46.27 | 0.44 | GC |
| GC50 | MOL004912 | Glabrone | 52.51 | 0.5 | GC |
| GC51 | MOL004913 | 1,3-dihydroxy-9-methoxy-6-benzofurano[3,2-c]chromenone | 48.14 | 0.43 | GC |
| GC52 | MOL004914 | 1,3-dihydroxy-8,9-dimethoxy-6-benzofurano[3,2-c]chromenone | 62.9 | 0.53 | GC |
| GC53 | MOL004915 | Eurycarpin A | 43.28 | 0.37 | GC |
| - | MOL004917 | Glycyroside | 37.25 | 0.79 | GC |
| GC54 | MOL004924 | (-)-Medicocarpin | 40.99 | 0.95 | GC |
| GC55 | MOL004935 | Sigmoidin-B | 34.88 | 0.41 | GC |
| GC56 | MOL004941 | (2R)-7-hydroxy-2-(4-hydroxyphenyl)chroman-4-one | 71.12 | 0.18 | GC |
| GC57 | MOL004945 | (2S)-7-hydroxy-2-(4-hydroxyphenyl)-8-(3-methylbut-2-enyl)chroman-4-one | 36.57 | 0.32 | GC |
| GC58 | MOL004948 | Isoglycyrol | 44.70 | 0.84 | GC |
| GC59 | MOL004949 | Isolicoflavonol | 45.17 | 0.42 | GC |
| GC60 | MOL004957 | HMO | 38.37 | 0.21 | GC |
| GC61 | MOL004959 | 1-Methoxyphaseollidin | 69.98 | 0.64 | GC |
| GC62 | MOL004961 | Quercetin der. | 46.45 | 0.33 | GC |
| GC63 | MOL004966 | 3'-Hydroxy-4'-O-Methylglabridin | 43.71 | 0.57 | GC |
| GC64 | MOL000497 | Licochalcone a | 40.79 | 0.29 | GC |
| GC65 | MOL004974 | 3'-Methoxyglabridin | 46.16 | 0.57 | GC |
| GC66 | MOL004978 | 2-[(3R)-8,8-dimethyl-3,4-dihydro-2H-pyrano[6,5-f]chromen-3-yl]-5-methoxyphenol | 36.21 | 0.52 | GC |
| GC67 | MOL004980 | Inflacoumarin A | 39.71 | 0.33 | GC |
| GC68 | MOL004985 | Icos-5-enoic acid | 30.70 | 0.20 | GC |
| GC69 | MOL004988 | Kanzonol F | 32.47 | 0.89 | GC |
| GC70 | MOL004989 | 6-prenylated eriodictyol | 39.22 | 0.41 | GC |
| GC71 | MOL004990 | 7,2',4'-trihydroxy－5-methoxy-3－arylcoumarin | 83.71 | 0.27 | GC |
| GC72 | MOL004991 | 7-Acetoxy-2-methylisoflavone | 38.92 | 0.26 | GC |
| GC73 | MOL004993 | 8-prenylated eriodictyol | 53.79 | 0.40 | GC |
| GC74 | MOL004996 | Gadelaidic acid | 30.70 | 0.20 | GC |
| GC75 | MOL000500 | Vestitol | 74.66 | 0.21 | GC |
| GC76 | MOL005000 | Gancaonin G | 60.44 | 0.39 | GC |
| GC77 | MOL005001 | Gancaonin H | 50.10 | 0.78 | GC |
| GC78 | MOL005003 | Licoagrocarpin | 58.81 | 0.58 | GC |
| GC79 | MOL005007 | Glyasperins M | 72.67 | 0.59 | GC |
| GC80 | MOL005008 | Glycyrrhiza flavonol A | 41.28 | 0.6 | GC |
| GC81 | MOL005012 | Licoagroisoflavone | 57.28 | 0.49 | GC |
| - | MOL005013 | 18α-hydroxyglycyrrhetic acid | 41.16 | 0.71 | GC |
| GC82 | MOL005016 | Odoratin | 49.95 | 0.30 | GC |
| GC83 | MOL005017 | Phaseol | 78.77 | 0.58 | GC |
| GC84 | MOL005018 | Xambioona | 54.85 | 0.87 | GC |
| GC85 | MOL005020 | Dehydroglyasperins C | 53.82 | 0.37 | GC |

Note: “-” Represent compounds of QHSSD without relative target.

Abbreviations: QHSSD (Qianghuo Shengshi decoction), OB (Oral bioavailability), DL (Drug-likeness), QH (Qianghuo), DH (Duhuo), GB (Gaoben), FF (Fangfeng), CX (Chuangxiong), MJZ (Manjingzi), GC (Gancao)


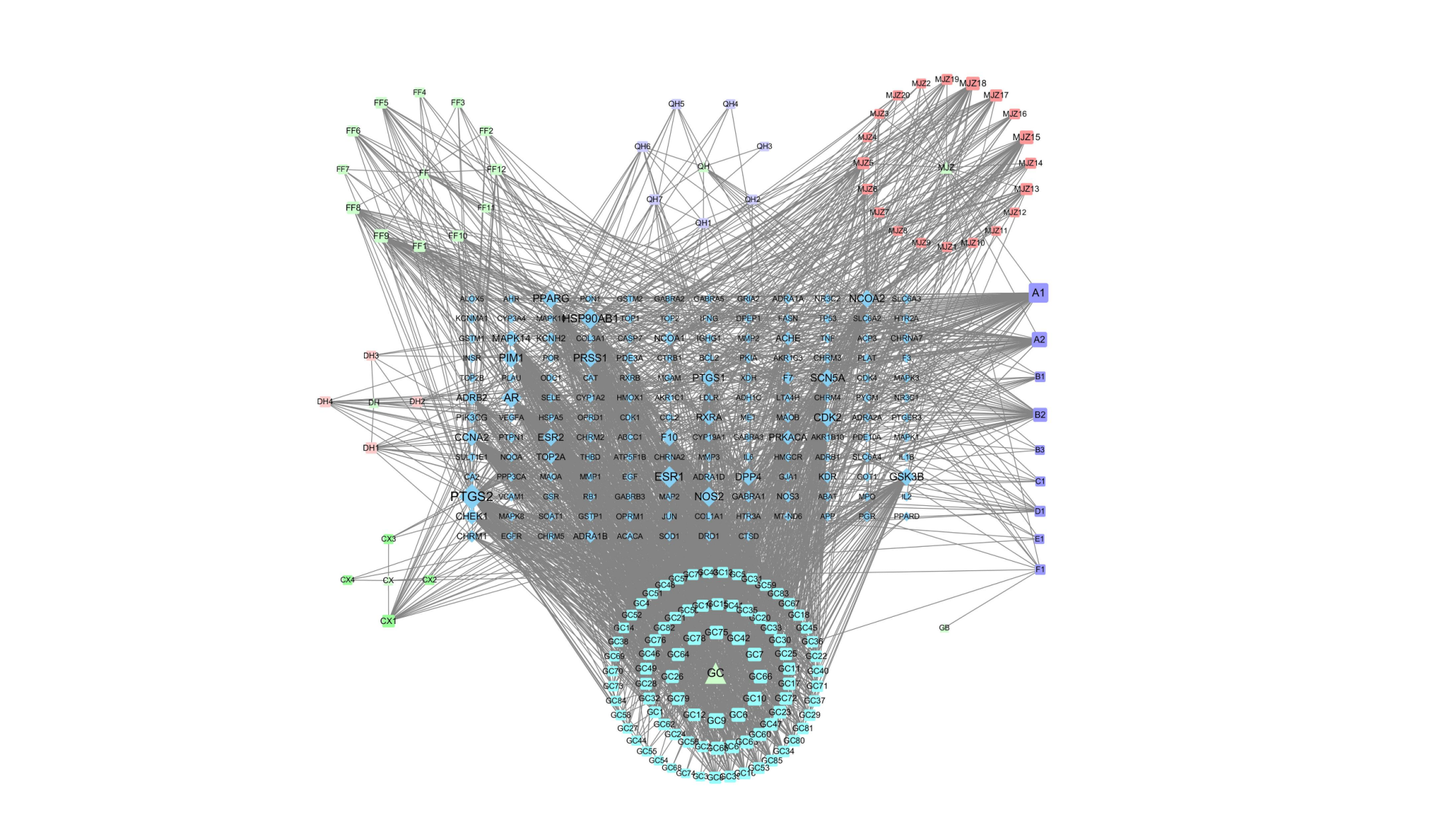


FIGURE S1: Herb-Compound-Target network of QHSSD. (The triangle nodes-the herbs of QHSSD, the rectangle nodes-the compounds of herbs, the diamond nodes-the targets of compounds)
